# Does method matter? Assessing the validity and clinical utility of structured diagnostic interviews among a clinical sample of first-admitted patients with psychosis: A replication study

**DOI:** 10.3389/fpsyt.2023.1076299

**Published:** 2023-04-03

**Authors:** Erling Inge Kvig, Steinar Nilssen

**Affiliations:** ^1^Nordland Hospital Trust, Bodø, Norway; ^2^UiT The Arctic University of Norway, Tromsø, Norway

**Keywords:** structured interview, diagnosis, schizophrenia spectrum, psychopathology, phenomenology

## Abstract

**Introduction:**

Increasingly, diagnostic assessments in clinical practice are made using structured diagnostic interviews or self-rating scales imported into clinical practice from research studies and big-scale surveys. Although structured diagnostic interviews have been shown to be highly reliable in research, the use of such method in clinical contexts are more questionable. In fact the validity and clinical utility of such methods in naturalistic contexts have rarely been evaluated. In this study we report on a replication study of Nordgaard et al (22) *Assessing the diagnostic validity of a structured psychiatric interview in a first-admission hospital sample*. World Psychiatry, 11 (3): 181–185.

**Methods:**

The study sample comprises 55 first-admitted inpatients to a treatment facility specializing in the assessment and treatment of patients with psychotic disorders.

**Results:**

We found poor agreement between diagnoses generated by Structured Clinical Interview for DSM-IV and Best-estimate consensus diagnoses (κ value 0.21).

**Discussion:**

We identified over-reliance on self-report, vulnerability to response set in dissimulating patients, and a strong diagnosis and comorbidity focus, as possible reasons for misdiagnosis with the SCID. We conclude that structured diagnostic interviews performed by mental health professionals without solid psychopathological knowledge and experience are not recommendable for clinical practice.

## 1. Introduction

Structured diagnostic interviews have rapidly become the gold standard in psychiatric research and diagnostic assessments in clinical practice. Originally developed for use in big-scale surveys and research studies, their recommendations by textbooks in psychiatry (1) and clinical guidelines (2) have made the use of these diagnostic tools widespread also in routine diagnostic assessments.

The historical foundation of the structured interviews was the traditional “mental status” examinations developed in European clinical psychiatry [e.g., Kraepelin (3)], and introduced into American psychiatry by Adolph Meyer in 1917 (4). Although numerous approaches for evaluating mental status have existed since (5), the Mental Status Evaluation Record (MSER) (6) have been influential to later structured diagnostic interviews in general and to the subsequent development of Schedule of Affective Disorders and Schizophrenia [SADS, (7)] and Structured Clinical Interview for DSM [SCID, (8)] (9).

Structured diagnostic interviews were introduced to improve diagnostic reliability (10). The well-known United States-United Kingdom diagnostic project demonstrated very different diagnostic habits among British and American clinicians (11). A solution to such reliability concerns, proposed among others by Robert Spitzer in the polemic paper stating “Are clinicians still necessary?,” was to minimize variance by standardization of psychiatric assessment and diagnostic criteria (12). The purpose of a structured interview is to provide a systematic evaluation by standardizing (1) the specific language of clinical inquiries, (2) the sequencing of these inquiries, and (3) the quantification of responses (9). With the «operational revolution» and the publication of DSM-III, including diagnostic criteria for most mental disorders, structured interviews such as the SCID were developed to standardize DSM-III evaluations of mental disorders. While the previous MSE-based structured interviews were organized around symptoms or topic areas (e.g., observed psychopathology, speech and thought, etc.) to provide comprehensive examination of psychopathology, the SCID is organized by diagnostic categories to provide adequate coverage of DSM inclusion criteria across a broad range of mental disorders.

The specific design of the SCID was developed to reduce variability in diagnostic assessments. Information variance, i.e., variability in the quality and quantity of information elicited across patients, is reduced by defining initial clinical inquiries as obligatory questions, with specification of further branching and probing questions. Inquiries are sequenced in a predefined order to assure adequate coverage of DSM criteria for specific diagnostic categories. Criterion variance, i.e., variability in converting clinical information into diagnostic criteria, is reduced by operational definitions of what is clinically relevant and when criteria are met. Most items are worded in the pathological direction (i.e., affirmative responses signifies impairment), and can be answered with a “yes” or “no.”

The SCID has been found to yield reliable diagnoses for most axis I disorders in research contexts (13–19). However, the validity and clinical utility of structured diagnostic interviews have rarely been explored (20, 21). Validity pertains to the actual correctness and usability of a test, while clinical utility concerns the specificity and sensitivity of tests. In psychiatric research, validity is often explored by examining the psychometric properties inherent to a diagnostic test. In a study by Nordgaard et al. (22), diagnostic validity was examined through measures of agreement and accuracy when compared to a criterion or “gold standard” (i.e., a well validated and generally accepted method). Best estimate consensus diagnoses was used as “gold standard” in the study. In the best estimate consensus procedure, interview data are supplemented with all existing sources of information (e.g., cross-sectional and longitudinal self-report data, observations by clinical staff in inpatient settings, longitudinal data from medical records, judgments from other clinicians, interviews of informants, and other sources) and evaluated by several experienced clinicians, who assign a best-estimate consensus diagnosis. Using the best-estimate consensus procedure as “gold standard,” Nordgaard et al. (22) found overall very low agreement between SCID derived and best-estimate consensus diagnoses (kappa 0.18), indicating major problems with the accuracy of diagnoses based on SCID in clinical settings. Since treatment decisions usually are based on the diagnosis, such diagnostic problems may have profound consequences for the patients. The interpretation of these findings was that, although standardization of psychiatric interviews may minimize information and criterion variability, this does not guarantee the quality (validity) of the information. The authors conclude that structured diagnostic interviews administered by mental health professionals without adequate psychopathological knowledge and experience are not recommendable for clinical work.

The recommendation of the use of structured diagnostic interviews as best standard by the Norwegian health authorities (2) provided an opportunity to investigate the validity and clinical utility of structured interviews as used in routine characterization of mental health patients in a naturalistic setting. In the current study we aimed to replicate the study by Nordgaard et al. (22), and examine the diagnostic validity and clinical utility of a structured diagnostic interview in a naturalistic observational study. We were interested in the following questions:

1. What is the sensitivity, specificity and validity of SCID-generated diagnoses compared with best-estimate consensus diagnoses?
2. What are the reasons for potential discrepancies found between SCID and best estimate consensus diagnoses?

## 2. Method

### 2.1. Setting

The study was carried out in Northern Norway (population 480,000). Health care is provided by a two-tier public health system, where specialist referrals are made by GPs and emergency clinics in the local municipalities with one or several GPs on duty. Specialist mental health care is provided by psychiatric departments in general hospitals, and in community mental health care centers. A specialist inpatient unit (Regional Unit of Psychosis) for patients needing specialist assessment and treatment for psychotic disorders is located in the general hospital located in Bodø (Nordland hospital), the administrative center for one of the larger counties in Northern Norway.

### 2.2. Subjects

The study population was consecutive patients admitted to Nordland hospital, Regional Unit of Psychosis. Patients were eligible for inpatient care at the unit if they were between 16 and 65 years and presented with near psychotic or psychotic symptoms as assessed by their referring GP or treating clinician in community mental health or local acute wards. None of the patients were acutely ill. If patients were too unwell at first contact, they were transferred to the acute ward for initiation of treatment, and assessed when transferred back again to the unit. As part of routine assessment patients were given a systematic assessment battery described below as “comprehensive diagnostic assessment.” With the publication of new national guidelines for patients with psychotic disorders in 2013, recommending the use of structured diagnostic interviews, the SCID-I/P (23) was added to routine assessments. This part of the routine assessments is described below as “structured diagnostic assessment.” This assessment was conducted by a specifically trained social worker independent from the clinical team of the patient. The use of a trained social worker and not an experienced psychiatrist or psychologist for the structured diagnostic interviews is similar to how such tools are typically used in research and in clinical practice, and is in line with both the SCID User’s guide (24) and research on structured diagnostic interviews showing no substantial differences in diagnosis made by mental health professionals without background in psychopathology and experienced clinicians (25). All patients were informed that quality control procedures were an integrated part of the treatment program and that all assessments were part of the standard quality control instruments of the health service offered at the Regional unit of Psychosis at Nordland hospital. This paper uses data collected as part of the routine assessment procedure at the unit. The study was approved by the Nordland hospital Data Protection Officer (Personvernombudet), 12 December 2021.

### 2.3. Data collection

The structured diagnostic interview was conducted by a social worker with clinical experience with severely ill patients, but with no previous training in psychopathology. The interviewer was specifically trained according to the SCID User’s guide (24, 26) and the SCID-101 didactic video course. Before implemented in routine assessment, two training interviews, observed and supervised by an experienced clinical psychologist, were conducted. The structured interview protocol consisted of the SCID-I/P (23) and the Schizotypal Personality Disorder module from SCID-II. The interview was conducted in a fully structured way with the following parts:

1. SCID-I/P Overview: an open-ended overview of the present illness and past episodes. Information on prior treatment, social and occupational functioning, context of the development of symptoms was also collected.
2. SCID-I/P Screening module: containing 12 screening questions where positive answers are followed up in relevant SCID modules later in the interview.
3. SCID-I/P Module A-J: contains separate modules for Mood episodes, Psychotic and associated symptoms, Psychotic disorders, Mood disorders, Substance Use disorders, Anxiety disorders, Somatoform disorders, Eating disorders, Adjustment disorders, Optional module. All relevant modules were completed.

The interviewer asked the mandatory pre-formulated questions in the predefined order, and used additional probes and questions for more information as specified in the SCID-I/P protocol and User’s guide. The average length of the interview was 1.5 h. Allocation of diagnoses, using the SCID-I/P Summary score sheet, was supervised by an experienced clinical psychologist in each individual case.

The comprehensive diagnostic interview was conducted by a treating clinician at the unit and the clinician was either an experienced psychiatrist or clinical psychologist. The clinical data was collected in phenomenologically-oriented, semi-structured interviews, modeled after the original study by Nordgaard et al. (22), with two mutually intertwined parts, and subsequently scored on a composite checklist.

1. Mental State Examination: a systematic examination of expressive features (e.g., affect modulation, contact quality, gaze, stereotypes, mannerisms, disorganization, and disorders of language), and objective evaluation of symptoms (e.g., mood, perceptual disturbances, thought content, and cognitive symptoms).
2. Anamnesis, psychosocial history and psychopathology: a detailed history was obtained to provide trajectory and context to further psychopathological examinations, also allowing a conversational interview according to the principles of phenomenological psychopathology. The psychosocial history was complemented with thorough review of medical records and collateral information from relatives.

Data was also collected through routine physical and psychological investigations, including a general physical examination, orienting neurology, blood and lab tests, and psychological investigations of cognition and personality.

Interviews were conducted according to the phenomenological principles proposed by Jaspers and others (27–30). The comprehensive diagnostic interviews began with a detailed and chronological, psychosocial history, providing opportunities for observing expressive features such as thought disorders and negative symptoms, and served as a natural point of departure for specific psychopathological exploration by asking for more details and examples. Psychopathology was explored and evaluated within the context of the patient’s life history and phase of illness. The style was free, dynamic and conversational, where questions or requests for more information were adapted to the patient’s narrative. The structure of the interview relied on the obligation of the interviewer to score all items on a composite checklist, the Operational Criteria Checklist [OPCRIT, (31, 32)], expanded with additional items Schedule for Affective Disorders and Schizophrenia (7), and the Examination of Anomalous Self-Experience (33).

Allocation of diagnoses was done using a best-estimate consensus procedure (34). All data from routine assessments, symptom ratings, referral letters, medical records, collateral information, and para-clinical data was presented in a case conference to a multi-disciplinary team, consisting of several experienced psychiatrists and clinical psychologists. Any differences on symptom criteria were resolved through consensus discussions. A best-estimate diagnosis was allocated to each patient using the OPCRIT system according to the ICD-10 and DSM-IV classification systems. In this study, we compared DSM-IV best-estimate consensus diagnoses to SCID diagnoses.

### 2.4. Data analysis

Unweighted kappa statistics was used to estimate agreement between the two diagnostic procedures for multiple- and dichotomized diagnostic categories. Cohen’s kappa indicates the proportion of agreement beyond that expected by chance. A kappa value that equals 0 indicates that the observed agreement is equal to the chance agreement. As suggested by Landis and Koch (35), the strength of agreement beyond chance for different κ values is Poor (<0), Slight (0–0.20), Fair (0.21–0.40), Moderate (0.41–0.60), Substantial (0.61–0.80), and Almost perfect (0.81–1.00).

Sensitivity and specificity was calculated using 2 × 2 tables that indicate number of false positives and negatives and true positives and negatives. Sensitivity estimates the proportion of persons with the disorder that will be correctly identified, while specificity estimates the proportion of persons without the disorder that will be correctly identified. Sensitivity and specificity values, using best-estimate consensus procedure as gold standard, were calculated for the SCID for multiple- and dichotomized diagnostic categories.

For the purpose of assigning a main diagnosis, we imposed the following hierarchy on the DSM-IV diagnoses (following the hierarchy of ICD-10): (1) schizophrenia, (2) other (non-affective) psychoses, (3) schizotypal disorder, (4) bipolar illness, (5) major depression, (6) other diagnoses (anxiety disorders, adjustment disorder, substance abuse/dependence). If a patient fulfills diagnostic criteria for several diagnoses, then the disorder placed highest in the hierarchy outranks lower placed diagnoses.

Data were analyzed using SPSS (version 24) for Macintosh (Table 1).

**Table 1:** DSM-IV diagnoses made by a trained non-clinician using Structured Clinical Interview for DSM-IV (SCID-I/P) vs. those made on the basis of a Best-estimate consensus procedure.

SCID diagnoses	Best estimate consensus diagnoses								
		Schizophrenia	Non-affective psychosis	Schizotypal disorder	Major depression	Bipolar	Other	Deferred diagnosis	Total
	Schizophrenia	16	0	0	0	0	1	0	17
	Non-affective psychosis	3	1	0	0	0	0	0	4
	Schizotypal disorder	0	0	0	0	0	0	0	0
	Major depression	2	2	3	1	1	0	0	9
	Bipolar	1	0	0	0	0	0	0	1
	Other	6	0	2	1	0	4	0	13
	Deferred	7	0	1	0	0	2	1	11
	Total	35	3	6	2	1	7	1	55

## 3. Results

Participants in this study were patients receiving inpatient care between 1 August 2014 and 1 August 2016. The total sample was 74 patients. Of these, 19 patients were excluded from the study sample. Reasons for exclusion were: (1) they were not first-admission patients (*n* = 11), (2) they were discharged before assessment could be initiated (*n* = 6), or (3) they could not participate in assessments because of language problems or did not cooperate with assessment (*n* = 2). The final study sample consisted of 55 patients (*n* = 55), comprising 43 men (78%) and 12 women (22%). Mean age at entry of the study was 25 years (range 16–40).

All patients were interviewed several times during the first phase of their inpatient stay for the comprehensive diagnostic assessment, and subsequently with the structured diagnostic assessment. Average length of stay was 17 weeks (range 3–67). Mean time from admission to the structured diagnostic interview was 41 days (range 5–230).

Cross tabulation of DSM-IV diagnoses by the two diagnostic procedures appear in Table 2. Kappa of overall concordance between the diagnostic procedures was 0.21. The kappa agreement between SCID and best-estimate diagnoses with the sample dichotomized into the schizophrenia spectrum (schizophrenia, other psychoses, schizotypal disorder) vs. non-spectrum (the remaining diagnostic groups combined) was 0.20.

**Table 2:** SCID diagnoses and number of comorbid diagnoses.

SCID diagnoses	SCID number of comorbid diagnoses					
		NCD^1^	+1	+2	+3 or more	Total
	Schizophrenia	0	9	4	4	17
	Non-affective psychosis	0	3	0	1	4
	Major depression	0	2	6	1	9
	Bipolar	0	1	0	0	1
	Other	0	2	5	6	13
	Deferred	11	0	0	0	11
	Total	11	17	15	12	55

1 NCD, no comorbid diagnoses.

+1, one comorbid diagnosis, +2, two comorbid diagnoses, +3 or more, three or more comorbid diagnoses.

Using the best estimate consensus diagnoses as gold standard, the sensitivity and specificity of the SCID for schizophrenia alone were 46% and 95%, respectively. The figures for the schizophrenia spectrum (schizophrenia, other non-affective psychoses and schizotypal disorder) were 45.5% and 91%.

In 33 (60%) of the 55 patients, the SCID diagnosis differed from the best-estimate consensus diagnosis. The best-estimate consensus procedure identified more patients with a schizophrenia spectrum diagnosis than the SCID procedure, i.e., 35 vs. 16, respectively. In addition, SCID classified all patients with a best-estimate consensus diagnosis of schizotypal disorder (*n* = 6) with other or no diagnosis. In explorative analysis, using numerical summaries of the data and visualizations, we identified two possible explanations for these disagreements.

1. Incomplete information: Among the 19 non-identified schizophrenia patients by SCID, seven had a deferred diagnosis, six received other diagnoses such as substance abuse/dependence (*n* = 4) or anxiety disorder (*n* = 2), three patients were diagnosed with an affective disorder, while three received a diagnosis of other psychoses (delusional disorder or psychosis NOS).
2. Level of comorbidity: The SCID diagnostic procedure yielded high levels of comorbidity. The number of comorbid diagnoses in addition to the main diagnosis is shown in Table 2. In 27 patients (49%), the SCID generated two or more comorbid conditions. The diagnostic category “Major depression” was the most common diagnosis identified with the SCID, and 19 patients (35%) received this diagnosis either as the main diagnosis or as a comorbid diagnosis.

## 4. Discussion

We found low overall kappa values (κ value 0.21), and slightly lower when comparing spectrum level diagnoses (κ value 0.20). This is in line with the finding of Nordgaard et al. (22), who found comparable low kappa values (κ value 0.18). According to the standard used in the literature on kappa statistics, this corresponds to a category of «questionable agreement» (35, 36). In terms of the clinical utility estimates, we found that the SCID procedure failed to detect 24 out of 44 patients with a schizophrenia spectrum diagnosis (a sensitivity of 45.5%). These findings corroborate those by Nordgaard et al. (22) and point to major problems in applying SCID to clinical settings.

Studies comparing structured diagnostic interviews with other diagnostic procedures have sometimes found better performance than our findings by the structured interviews with kappa values reported between 0.59–0.90 (20, 37, 38). However, in all of these studies, variations in the sources of information used in the compared diagnostic procedures are of great importance. In the study by Basco et al. comparing expert-generated diagnosis with SCID interviews performed by trained psychiatric nurses, (37), an important finding was that SCID generated diagnoses significantly improved with additional data sources such as review of medical records. In studies finding high levels of agreement between SCID generated research diagnosis and routine clinical diagnoses, the SCID diagnoses are reported to be based on interview data and reviews of medical records (39, 40). Indeed, in the study by Fennig et al. (41), SCID elicited data was found to be an incomplete source of data in the detection of psychosis in a first-admission sample. In their sample of first-admission patients with psychosis, 13.7% denied all their psychotic symptoms, while 33.3% revealed only some of their symptoms. The conclusion was that SCID generated diagnoses without incorporating longitudinal and external sources of clinical data, and the generated diagnoses were therefore of questionable validity.

The explorative analysis in the current study highlighted that many patients with a best-estimate consensus diagnosis of schizophrenia spectrum, either received no diagnosis (deferred diagnosis) or other non-psychotic diagnoses such as anxiety, depression or substance abuse. There may be several explanations for this. It is possible that many patients seen in clinical practice tend to dissimulate their psychotic experiences, and the SCID is particularly vulnerable to such response styles. Thus, the use of structured diagnostic interviews, developed in research contexts where participants have consented to take part in a research study, in clinical practice may lead to an over-reliance on the patients willingness to disclose their innermost experiences. This is possibly also compounded with the ways most questions are phrased in structured interviews. In an effort to reduce criterion variance, clinical inquiries in the SCID are designed to be unidirectional where endorsement of a question signifies psychopathology. This transparency, where the patient has a very clear idea of the purpose of questions and is free to modify answers accordingly, may lead to a response set where all psychotic symptoms are denied, leading to either no diagnosis or a non-psychotic diagnosis (9). Another possibility is that the phrasing of many questions in the SCID is very specific, often simply a rephrasing of diagnostic criteria, and some patients may not recognize their own experiences as instances of the symptom. Thus, a patient may deny having thought insertion in response to the question “Do you ever experience certain thoughts that are not your own are being placed in your head?,” could still harbor experiences of alien thoughts, not necessarily “being placed” or “inserted” into his mind, but perhaps rather experienced as simply being there (42).

We also found high levels of comorbidity in the SCID generated diagnoses. This is in part a consequence of the explicit encouragement by the DSM system to make as many diagnoses as necessary to cover the full clinical picture. A similar formulation can be found in ICD-10 but it has a different implication in terms of recorded diagnosis due to its diagnostic hierarchy, which, by contrast, is not present to the same extent in DSM. Thus, rather than focusing on the exploration of symptomatology, the SCID explicitly focuses on diagnoses. This is reflected in the clinical inquiries, where the predetermined questions are presented in a definite order (43), the order being specified by the diagnostic criteria listed in the DSM manual. By contrast, in clinical interviews taking the chronological, psychosocial history as a point of departure for psychopathological exploration, symptoms and signs are more naturally evaluated in the context of the patient’s life history and phase of illness. Rather than evaluating symptoms and signs as related to possible co-occurring disorders, careful exploration of the progression of psychopathology reveal many of these may be psychopathological aspects of the same disorder. In the schizophrenia spectrum, most patients report experiences of depression and anxiety in the course of illness. However, these experiences are not co-occurring independent disorders but are well-known aspects of schizophrenia and should therefore, in ICD-10, typically not be recorded as comorbid diagnoses to the index diagnosis of schizophrenia (44). Psychopathological phenomena are generally interdependent aspects of a whole or a “gestalt” (45). In phenomenological psychopathology, a «psychopathological gestalt» is a «certain unifying structure of experimental, expressive and behavioral phenomena, which transpires through, connects, shapes and colors the symptoms and signs that may occur within a given mental disorder» (46). Multiple nonpsychotic symptoms, perhaps with a pervasive pattern considered atypical of true neurotic disorders conveyed by the classical notion of “pseudoneurotic symptoms” (47), should cause suspicion of a common psychopathological process. But such gestaltic considerations is less likely in criteria-focused structured diagnostic interviews. Preformed questions and the strict sequencing of clinical inquiries, do not allow the “staying with” psychopathological experiences and withholding interpretation in terms of symptoms and signs, until the experience is fully grasped.

There are some limitations to the generalizability of these findings. The sample size is small, we used only one interviewer for the structured interviews, and the participants were not randomly selected from a general, heterogenous psychiatric population. Participants were all first-time admitted patients and referral was based on evaluation of the presence of psychosis. The study group included a larger proportion of patients with a schizophrenia spectrum diagnosis, making the comparison to other more heterogenous samples uncertain. We do not believe that these limitations seriously question the main findings. One of the strengths is that the study sample is from an ordinary specialist health care responsible for delivering care to patients in a catchment area of 480,000, which makes the ecological relevance high.

The implications of this study for recommendations given in clinical guidelines and the training of clinicians are significant. In clinical practice, accurate diagnosis is of immense value for patients. Clinicians have practical concerns, and diagnoses are crucial for treatment planning and treatment decisions. Patients who are misdiagnosed may receive inadequate or ineffective treatment, and risk readmission with diagnostic reassessments, often with different or even incompatible diagnoses. Using ineffective diagnostic methods in mental health services is costly in terms of the time and resources spent.

Our finding that a structured diagnostic procedure, at least as performed in a fully structured way without solid psychopathological knowledge and without utilizing all available sources of information, is not an adequate way of allocating diagnoses in clinical practice. It is likely that an experienced clinician with training in psychopathology, taking advantage of the possibility to ask further questions and availability of other sources of information, would arrive at more valid diagnoses. Indeed, according to the User’s guide (24), it is allowed to use the SCID as a diagnostic checklist rather than a structured interview, with information obtained from other sources. However, as we argue, some of the problems with structured diagnostic interviews are inherent to the criteria-focused format of the clinical inquiries in the structured methods. This makes it difficult for clinicians using a structured diagnostic interview to make considerations of the gestalt when exploring psychopathology. These considerations are crucial for adequate differential-diagnosis.

Overall, there is a need to revive teaching and training in psychopathology and comprehensive differential-diagnosis. The most important factor for successful interviewing is knowledge of psychopathology. This requires far more than training in a specially selected interview schedule. Training in psychopathology implies academic studies, supervised diagnostic interviews, joint interviews with colleagues, ongoing peer-discussions of psychopathological concepts, and participation in consensus meetings with experts for differential-diagnostic evaluations.

In conclusion, the findings in the current study are largely in agreement with the findings in Nordgaard et al. (22), and it adds further support to their claim that structured diagnostic interviews performed by mental health professionals without solid psychopathological knowledge and experience, is not recommendable for clinical practice.

## Data availability statement

The raw data supporting the conclusions of this article will be made available by the authors, without undue reservation.

## Ethics statement

Ethical review and approval was not required for the study on human participants in accordance with the local legislation and institutional requirements. Written informed consent from the participants’ legal guardian/next of kin was not required to participate in this study in accordance with the national legislation and the institutional requirements.

## Author contributions

EK was the primary investigator, did the data analysis, wrote the outline and first draft of the manuscript, and is responsible for the overall content as guarantor. EK and SN designed and planned the study and were actively involved in interpretating the results, revising the manuscript and approving the final version. All authors contributed to the article and approved the submitted version.

## Conflict of interest

The authors declare that the research was conducted in the absence of any commercial or financial relationships that could be construed as a potential conflict of interest.

## Publisher’s note

All claims expressed in this article are solely those of the authors and do not necessarily represent those of their affiliated organizations, or those of the publisher, the editors and the reviewers. Any product that may be evaluated in this article, or claim that may be made by its manufacturer, is not guaranteed or endorsed by the publisher.
